# Green tea as a cosmetic agent for skin aging: A scoping review

**DOI:** 10.22038/ajp.2025.25449

**Published:** 2025

**Authors:** Ratih Puspita Febrinasari, Benedictus Benedictus, Kenneth Tan, Yasmine Mardhiati, Salsabilla Kania Putri, Syaiful Choiri, Dinar Sari Cahyaningrum Wahyuni

**Affiliations:** 1 *Departement of Pharmacology, Faculty of Medicine, Universitas Sebelas Maret, Surakarta, Indonesia*; 2 *Department of Medicine, Faculty of Medicine, Universitas Sebelas Maret, Surakarta, Indonesia*; 3 *Department of Pharmacy, Faculty of Mathematics and Sciences, Universitas Sebelas Maret, Surakarta, Indonesia*; 4 *Pharmaceutical Technology and Drug Delivery, Department of Pharmacy, Universitas Sebelas Maret, Surakarta, Indonesia*; 5 *Pharmaceutical Biology, Department of Pharmacy, Universitas Sebelas Maret, Surakarta, Indonesia*

**Keywords:** Green tea, Polyphenols, EGCG, Skin aging, Cosmetic

## Abstract

**Objective:**

Tea is known to have antioxidant and anti-inflammatory properties. Also, skin care products often contain antioxidant compounds that help protect the skin from free radicals that cause premature aging. In this study, we investigated the antioxidant and anti-aging effects of whole green tea (*Camellia sinensis)* and its polyphenols, especially EGCG (Epigallocatechin-3-gallate) on human skin.

**Materials and Methods:**

This scoping review followed PRISMA-ScR (Preferred Reporting Items for Systematic Reviews and Meta-Analyses Extension for Scoping Reviews) guidelines. The literature search was conducted using keywords: "Green tea", "*Camellia sinensis*", "cosmetics", "dermatology", and "topical". The literature search in this study included journals published from January 2013 to December 2023. This scoping review aimed to answer questions about the benefits of green tea or tea polyphenol extract in cosmetics for skin aging problems. Since the aim of this scoping review is to give a broad overview of the subject matter, review articles are included to give all possible insights into this topic.

**Results:**

We included twenty-one articles for qualitative analysis. The included studies consisted of six *in vitro* studies, nine reviews, and six controlled trials, with twelve studies investigating the effects of whole green tea, four studies focusing on its polyphenols, and five studies examining the compound EGCG.

**Conclusion:**

This review gave an overview of green tea extract as an anti-aging in vivo and in vitro studies. Further research on the use of molecular carriers and their application to human skin is needed.

## Introduction

Skin aging is a natural process that is influenced by intrinsic and extrinsic (photoaging) mechanisms. This aging process is marked by phenotypic changes in skin cells as well as changes in extracellular matrix components such as collagen, elastin, and proteoglycans (Zhang and Duan, 2018). These components provide firmness, elasticity, and hydration to the skin. Intrinsic skin aging is a chronological process of physiological changes. Extrinsic aging is a combination of intrinsic aging and accelerated degeneration caused by ultraviolet radiation (UV), ionizing radiation, air pollution, and other external environmental factors (Ganceviciene et al., 2012; Chen et al., 2020). UV radiation exposure causes oxidative stress which causes disorganization of collagen fibers, resulting in functional changes (Rinnerthaler et al., 2015; Ghori et al., 2020; Bocheva et al., 2021). Reactive Oxygen Species (ROS) stimulates matrix metalloproteinase (MMP) synthesis via the mitogen-activated protein kinase (MAPK) pathway which reduces collagen and elastin production in the extracellular matrix causing an inflammatory reaction (Amaro-Ortiz et al., 2014; Limtrakul et al., 2016; Gu et al., 2020). Skin aging is characterized by the accumulation of non-functional and disorganized elastic fibers throughout the dermis, resulting in changes such as loss of elasticity, and sagging of the skin (Haydont et al., 2019; Shin et al., 2019; McCabe et al., 2020). Physiological aging cannot be stopped, but extrinsic aging can be decelerated with the use of topical anti-aging agents. UV-B radiation causes increased production of ROS which can cause DNA damage and initiation of photo-oxidation, especially in the epidermis of the skin through augmentation of cellular ROS levels. This leads to an imbalance of skin antioxidants, hence, accelerating photoaging. UV-B also causes elastin degradation in fibroblasts, resulting in loss of skin elasticity (Kwon et al., 2019). Skin areas that often experience extrinsic aging are faces, necks, upper arms, and hands because of UV radiation exposure that causes rapid aging (Chaudhary et al., 2020). Skin layers thin by around 10% every 10 years, so the skin is easily irritated, brittle, and dry because the amount of proteoglycan production and natural moisturizing factor (NMF) decreases (Rittie and Fisher, 2015; Al Amin et al., 2018; Nanzadsuren et al., 2022).

Tea comes from the *Camellia sinensis* (L.) Kuntze species, utilizing the young leaves of the plant. Tea is widely cultivated in Southeast Asia as raw material for making traditional medicines. Furthermore, tea is known to be efficacious as an antioxidant, anti-inflammatory, anti-carcinogenic, and even as an antimicrobial agent (Khan and Mukhtar, 2013; Chen and Lin, 2015; Dalming et al., 2019; Fadhilah et al., 2021). Based on the level of oxidation and fermentation, tea is divided into three main groups, namely green tea, oolong tea, and black tea. Green tea is a type of tea that is processed without going through fermentation. Oolong tea goes through partial fermentation, while black tea goes through complete fermentation (Fadhilah et al., 2021; Zhao et al., 2022). The difference in processing causes green tea to have the highest content of polyphenolic compounds (Salman et al., 2022). The fermentation process causes catechin compounds and their derivatives such as epigallocatechin-3-gallate (EGCG), a polyphenolic compound to oxidize (Farhan, 2022). Green tea is produced by applying heat or steam to fresh leaves to inactivate the polyphenol oxidase enzymes. This enzyme is thermolabile, so the heating process can reduce its activity and prevent the enzymatic oxidation of catechins (Yan et al., 2020).

The main fraction of catechins in green tea is EGCG, epigallocatechin (EGC), epicatechin gallate (ECG), and epicatechin (EC) (Ahmad et al., 2014). EGCG contributes about 59% of all catechins (Singh et al., 2011). The functional and structural differences between these catechin compounds relate to the number of hydroxyl groups in the B-ring and the presence or absence of galloyl moieties (Cai et al., 2018; Rady et al., 2018). EGCG reduces UVB-induced inflammatory responses and infiltration of leukocytes in human skin (Kim et al., 2001).

Antioxidants are compounds that are useful for counteracting the presence of free radicals that cause aging (Lobo et al., 2010; Putri, 2021). Free radicals can damage fatty acids and eliminate elasticity as a result of oxidative stress caused by an imbalance of free radicals and antioxidants in cells (Phaniendra et al., 2015; Aguirre-Cruz et al., 2020; Ayda et al., 2022). Antioxidants play an active role in neutralizing free radicals, hence, dermal tissues are protected from oxidative damage. Therefore, skin care products always contain antioxidant compounds to prevent premature aging (Silva et al., 2019; Unsal et al., 2021). In this study, we investigated the antioxidant and anti-aging effect of green tea and its polyphenols, especially EGCG on human skin and identified all potentially relevant evidence around the effectiveness of green tea on skin aging.

## Materials and Methods

### Study design

This scoping review adopted the guidelines recommended by the Preferred Reporting Items for Systematic Reviews and Meta-Analyses extension for Scoping Reviews (PRISMA-ScR) (Tricco et al., 2018). This scoping review was designed to answer questions about the benefits of green tea or tea polyphenol extract in cosmetics for skin aging problems. This question included the information of population (humans, animals, or cells (i.e. *in vitro* studies of skin)), concept (green tea or polyphenol extract of green tea effect on skin aging), and context (topical application of green tea).

### Eligibility criteria

To fully represent the current evidence on green tea or green tea polyphenol extract, we included review articles and original research papers. All included papers followed several criteria including all experiments related to skin aging problems, using green tea or green tea polyphenol extract. The papers were excluded if they were not written in English or if the full text could not be found. Papers that focused on skin cancer and oral supplementation were also excluded. In addition, case reports and duplicated studies were excluded from this study. Given that this is a scoping review which aims to give a broad overview on this subject, review articles are included in this scoping review to provide all possible insights to this topic.

### Search strategy

**Figure 1:**
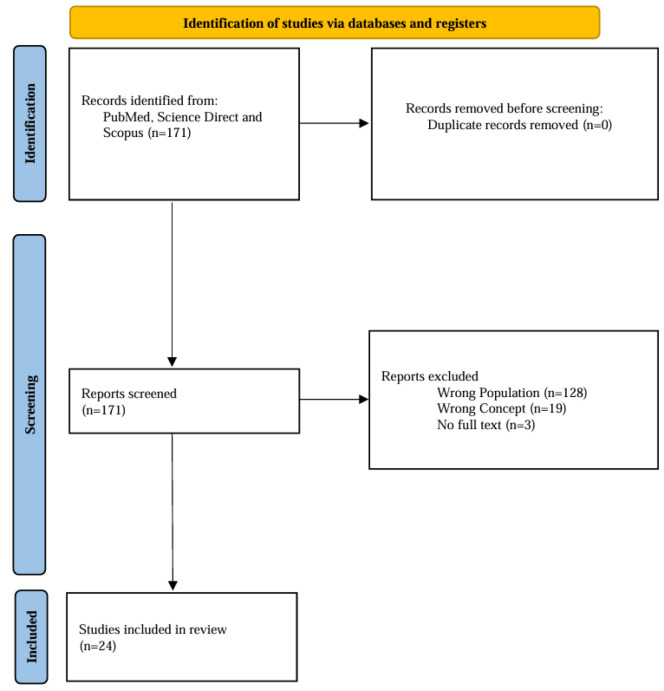
The PRISMA-Scr flowchart of study selection

The research samples were original or review articles published on three databases (PubMed, Science Direct, and Scopus). They were broadly searched from January 2013 until December 2023. The flowchart of this study is described in Figure 1. The literature search was conducted using the following keywords: *"Green tea", "Camellia sinensis", "cosmetics", "dermatology", *and* "topical"*. These keywords were used in combination for searching all databases to obtain relevant literature.

### Study selection and data extraction

Four authors independently screened the obtained studies. All disagreements that appeared were resolved by discussion. The inclusion of studies aligned with the population, concepts, and contexts that had been explained in the previous section. A summary of included journals and results from *in vitro* and trial studies are presented.

## Results

We searched PubMed, ScienceDirect, and Scopus for relevant literature and found 176 articles. None of the articles was a duplicate. We screened them by their titles and abstracts. Then, 133 articles that did not match our population criteria, nineteen articles that did not match our concept criteria, and three articles whose full text cannot be obtained, were excluded. We included the remaining twenty-one articles for qualitative analysis. The included studies consisted of six *in vitro* studies, nine reviews, and six controlled trial studies, with twelve studies investigating the effects of whole green tea, four studies focusing on its polyphenols, and five studies examining the compound EGCG (Abotorabi et al., 2020; Becker et al., 2019; Boo, 2019; Bowe and Pugliese, 2014; Ferzli et al., 2013; Frasheri et al., 2020; Gianeti et al., 2013; Hollinger et al., 2018; Hong et al., 2013; Hu et al., 2020; Juhász et al., 2018; Kim et al., 2019, 2018; Koch et al., 2019; Li et al., 2022; Lianza et al., 2020; Megow et al., 2017; Wagemaker et al., 2017; Wisuitiprot et al., 2022; Won et al., 2021; Zillich et al., 2015) The flow diagram of the selected study is presented in Figure 1, a summary of the findings from all results is presented in Table 1, and specific results for *in vitro* and trial studies are presented in Table 2.

**Table 1:** The summary of the studys’ findings of the effect from green tea as a whole and its polyphenols compounds

**References**	**Interventions**	**Study designs**	**Treatment durations**		**Dose**	**Route of administration**	**Population characteristics**	**Sample size**	**Age (years)**	**Key findings**
Kim et al.(2018)	EGCG	*In vitro* study	12-24 hour		12.5 and 25 µM	Parenteral route	HaCaT keratinocyte cell, RAW 264.7 macrophage cell, B16F10 melanin cell	N/A (cells as a sample of the population)	N/A (cells as a population)	EGCG has antioxidant activity and can improve skin moisture.
Kim et al. (2019)	3” Me-EGCG	*In vitro* study	24 ;48 ; and 72 hours		6,25 and 12,5 µM	Parenteral route	RAW264.7 cells, HaCaT cells (48h), HEK293 T cells (48 h)	N/A (cells as a sample of the population)	N/A (cells as a population)	3” Me-EGCG shows scavenging effects and antioxidant properties.
Won et al.(2021)	EGCG	*In vitro* study	45 minutes ; 24 hours		1, 10, 20 µM	Parenteral route	Hs68 cells	N/A (cells as a sample of the population)	N/A (cells as a population)	EGCG increases resistance to oxidative stress effect as an anti-aging.
Frasheri et al.(2020)	EGCG	Literature review	24 hours		N/A	N/A	N/A	N/A	N/A	EGCG has UV-protecting properties.
Li et al. (2022)	EGCG	*In vitro* study	24 hours		2 mg	Parenteral route	Human dermal fibroblast	N/A (cells as a sample of the population)	N/A (cells as a population)	EGCG-niosomes improve the antioxidant effect on skin cells.
Wagemaker et al.(2017)	green tea polyphenols	randomized controlled trial	4 days		527 µg/mL	Topical route	25 Japanese volunteers	25	24- 55 years	GTP accelerates skin cell recovery and injury healing.
Ferzli et al. (2013)	green tea polyphenols and caffeine	open label-controlled trial	12 weeks		2.5 mg	Topical route	Old males and females (n=32) having skin Fitzpatrick types II	16	35 years	Polyphenol inhibits UV damage.
Zillich et al.(2015)	polyphenols	Literature review	N/A		N/A	N/A	N/A	N/A	N/A	Polyphenolic extracts as a therapy for UV damage.
Boo (2019)	Phenolic compounds	Literature review	N/A		N/A	N/A	N/A	N/A	N/A	Phenolic compounds decrease ROS.
Koch et al.(2019)	green tea extracts	Literature review	N/A		N/A	N/A	N/A	N/A	N/A	Green tea extract as an anti-aging treatment.
Hollinger et al. (2018)	green tea	Systematic review	N/A		N/A	N/A	N/A	N/A	N/A	Green tea extracts inhibit UV damage effects.
Bowe et al (2014)	Green tea	Literature review		N/A	N/A	N/A	N/A	N/A	N/A	Tannase improves antioxidant activity.
Wisuitiprot et al.(2022)	Green tea extract encapsulated chitosan microparticles in emulsion	Randomized controlled trial		4-8 weeks	2.5 mg	Topical route	female volunteers have wrinkles on their faces.	29	25-55 years old	Green tea has an anti-aging effect.
Abotorabi et al. (2020)	jujube and green tea extracts	*In vitro* study		24 hour	0,5 mg/ml	Parenteral route	Human fibroblast cells	N/A (cells as a sample of the population)	N/A (cells as a population)	Green tea extract decreases in MMP-2 and MMP-9 at low concentrations.
Becker et al.(2019)	*Camellia sinensis* (tea) plants and catechin	Literature review		16 weeks	N/A	N/A	N/A	N/A	N/A	*Camellia sinensis* is safe to use in cosmetic products.
Hong et al.(2013)	tannase converted green tea treatment	Randomized Controlled Trial		8 weeks	2.5 mg	Topical route	Korean female volunteers have fine wrinkles at the lateral canthal lines and have skin Fitzpatrick types II and III	42	30-59 years	Tannase treatment improves the antioxidant activity of green tea.
Hu et al.(2020)	Green tea	Literature review		N/A	N/A	N/A	N/A	N/A	N/A	Green tea inhibits erythema caused by UV radiation.
Gianeti et al.(2013)	*Camellia sinensis* (green tea), glycolic leaf extract	Open label-controlled trial		30 days	6% (0.1 g/cm²)	Topical route	24 females have skin Fitzpatrick types II and III.	24	25-40 years	Green tea extracts have significant skin improvement effects.
Juhasz et al (2017)	Green tea	Literature review		N/A	N/A	N/A	N/A	N/A	N/A	Cosmeceuticals products of green tea have an antiaging effect.
Megow et al (2017)	Benifuuki and Yabukita green tea	Randomized Controlled Trial		2 weeks	3 cups tea	Oral route	No skin diseases	37 (21 women and 16 men)	20-55 years	Benifuuki tea contains more effective EGCG than Yabukita tea.
Lianza et al (2020)	*Camellia sinensis* (leaves)	*In vitro* study		24 hours	50 µg/ml	Parenteral route	Green tea extracts 50 µg/ml	50 µg/ml	N/A (using N-succinyl-Ala-Ala-Pro-Phe as a population)	*Camellia sinensis* can inhibit Elastase (39%) and Tyrosinase (43%).

Abbreviations: 3''Me-EGCG, (−)-Epigallocatechin-3-(3''-O-methyl) gallate; EGCG, Epigallocatechin Gallate; GTP, Green tea polyphenols; HaCaT, Cultured Human Keratinocyte; HEK293T, human embryonic kidneys 293T; Hs68, human dermal fibroblast; MMP, matrix metalloproteinase; RAW264.7, macrophage-like cell; ROS, reactive oxygen species; UV, ultra violet. N/A: not available

### Antioxidant effect of green tea and its ingredient, Epigallocatechin Gallate (EGCG) on human skin

#### In vitro studies

Green tea possesses antioxidant activity and anti-aging effects due to its secondary metabolites such as flavonoids, alkaloids, polyphenols, organic acids, amino acids, methylxanthine, volatile compounds, and other biological compounds (Rady et al., 2018; Putri et al., 2020). Green tea polyphenols (GTP) are the major compound that might play an important role in the antioxidant activity of green tea, especially in the catechin compound group and the derivatives. Dried tea leaves contain flavonoid compounds, one of which is catechin, at 42%, with the most abundant derivative compound being epigallocationchin-3-gallate (EGCG). (Wagemaker et al., 2017; Rabbani et al., 2020; Yan et al., 2020). 

Antioxidants from GTP are able to protect body cells from the harmful side effects of ROS which are related to the progression of skin aging (Zillich et al., 2015; Kwon et al., 2019). Previous studies showed that EGCG between 12.5 and 25 µM inhibited the effect of ROS production up to 50-60% after being exposed to skin-damaging substances like sodium nitroprusside (SNP) or dihydrorhodamine (DHR) and inflammatory factors like tumour necrosis factor-α (TNF-α) (Kim et al., 2018, 2019; Won et al., 2021). 

### Anti-inflammatory effect of green tea and its ingredients on human skin

#### In vitro studies

The protective effect of EGCG on ultraviolet light as the precursor of ROS production was found. Cell viability after UV-B exposure increased by 71-85% after the application of EGCG with the subgroup of EGCG, (−)-Epigallocatechin-3-(3''-O-methyl) gallate (3″Me-EGCG), having shown best result for UV-B protection effect (Kim et al., 2018, 2019; Abotorabi et al., 2020). Besides, another study suggested the protective effect of topically applied EGCG against acute skin damage caused by UV-A (Zillich et al., 2015). 

### The effect of green tea and its ingredients on elasticity and skin melanin index

#### In vitro studies

Free radical scavenging and cell proliferation promoting effect of green tea EGCG showed some promising results in reducing skin wrinkles and increasing skin elasticity (Hong et al., 2013; Megow et al., 2017; Kim et al., 2018, 2019; Wisuitiprot et al., 2022). *In vitro*, studies suggested that EGCG extract protected cells from free radical like 2,2-diphenyl-1-picrylhydrazyl (DPPH), 2,2′-Azino-bis (3 ethylbenzothiazoline-6-sulphonic acid) (ABTS), and hydrogen peroxide (H_2_O_2_) exposure. EGCG showed a protective effect of 40-85% from DPPH, 90% from ABST, and 80% from H_2_O_2_ (Kim et al., 2018, 2019). 

The proliferation of cells that received EGCG supplementation increased 200-310% during twenty-four hours and 500-600% during seventy-two hours in a dose-dependent fashion. The most optimal dose to increase this proliferating effect was achieved by supplementation of 25 µM EGCG (Kim et al., 2018, 2019). Other studies also showed the effect of green tea leaf extract to inhibit elastase, an enzyme that plays a role in skin elastin component degradation, up to 39% (Farage et al., 2008; Lianza et al., 2020; Marinaccio et al., 2022). Green tea extract, especially EGCG also elicited other promising effects to reduce melanin deposition and increase skin moisture (Wagemaker et al., 2017; Kim et al., 2018, 2019; Wisuitiprot et al., 2022). Furthermore, EGCG supplementation reduced melanin secretion by up to 70% and decreased melanin concentration by up to 50% compared to control cells. This effect was attained after supplementation of 100 µM EGCG, while a lower dose did not give any notable effects on melanin secretion or concentration (Kim et al., 2018). Other *in vitro* studies also showed the EGCG effect to reduce tyrosinase, the enzyme that is responsible for the hyper-pigmentation of skin, by up to 43% (Lianza et al., 2020; Slominski et al., 2004).

### Clinical trials

Some randomized controlled trial studies using topical and oral administration of green tea ensured the effect of green tea and the tannase-converted form of green tea scavenging effect from 2,2-diphenyl-1-picrylhydrazyl (DPPH), 3-ethylbenzothiazoline-6-sulfonic acid (ABTS), and H2O2-induced free radicals (Hong et al., 2013; Megow et al., 2017). The studies suggested that the converted form of tannase-green tea extract (TGE) was superior to normal green tea extract (NGE) with IC50 values are being presented after introduction of controlled trials respectively IC50 1.45 mg/ml vs 1.63 mg/ml (DPPH exposure, p<0.01) and IC50 1.44 mg/ml vs 0.98 mg/ml (ABTS exposure, p<0.001). Also, it is not clear in what model this data was obtained (Hong et al., 2013; Megow et al., 2017).

An open-label study in humans showed GTP combination with resveratrol and caffeine that were administered topically reduced UV-induced erythema and facial redness after 3-6 weeks of therapy without any notable side effects (Ferzli et al., 2013). This achieved effect can be from the result of EGCG's role in reducing MAPK phosphorylation that controls the expression of MMP-1, -2, and -9, via mitogen-activated protein extracellular kinase (MEK) - extracellular signal-regulated kinase (ERK) pathway, and regulation of nuclear factor-kappa B (NF-κB) expression (Kwon et al., 2019; Abotorabi et al., 2020; Won et al., 2021). 

**Table 2:** The Findings from *in vitro* and controlled trial study of green tea as a whole and its epigallocatechin gallate compounds

**Authors, years**	**Population or cells used**	**Assessed effects**	**Substances**	**Dose and Effects**	**Comparisons**	**Effects**
Kim et al, 2018	HaCaT keratinocyte cells	Toxicity	EGCG	EGCG up to 25 µM showed no toxicity	-	-
		Effects on skin hydration via natural moisturizing factors (NMF) synthesis.	EGCG (12.5 µM and 25 µM)	FLG was significantly increased by EGCG compared with retinol treatment. The levels of TGM-1, HAS-1, and HAS-2 were also augmented similar to the effect of retinol.	Retinol (10 µg/ml)	levels of TGM-1, HAS-1, and HAS-2 were augmented
		Cell proliferation	EGCG (12 hours)	240% for 12.5 µM EGCG and 265% for 25 µM EGCG from baseline	Retinol (10 µg/ml for 12 hours)	150% from baseline
			EGCG (24 hours)	269% for 12.5 µM EGCG and 310% for 25 µM EGCG from baseline	Retinol (10 µg/ml for 24 hours)	208% from baseline
		protective activity against UVB irradiation	EGCG	12.5 µM EGCG increased the cell viability to 72.8%, and 25 µM EGCG significantly increased viability to 75.9%.	HaCaT cells without additional substances	Cell viability was decreased to 68.9%.
		DPPH scavenging activity	EGCG	40% (6.25 µM EGCG), 60% (12.5 µM EGCG), and 85% (25 µM EGCG)	Ascorbic acid 500 µM	90%
		ABTS scavenging activity	EGCG	90% in 6.25 µM, 12.5 µM, and 25 µM EGCG -	Ascorbic acid 50 µM	90%
		NO production in 24 hours after 1.5 mM SNP induction	EGCG	80% (12.5 µM EGCG) and around 76% (25 µM EGCG)	HaCaT cells without additional substances	100%
		Cell viability after 24 hours	EGCG	70% (12.5 µM EGCG) and 80% (25 µM EGCG)	HaCaT cells without additional substances	60%

	RAW 264.7 macrophage cell	ROS generation after 0.25 mM SNP induces	EGCG	50% (12.5 µM EGCG) and 40% (25 µM EGCG)	RAW 264.7 cells without additional substances	80%
	B16F10 melanin cell	Toxicity	EGCG	EGCG up to 100 µM showed no toxicity.	-	-
		Melanin secretion	EGCG	120% (25 µM EGCG), 130% (50 µM EGCG), and 70% (100 µM EGCG) from control	Arbutin 1 mM	50% of control
		Melanin content	EGCG	100% (25 µM EGCG), 80% (50 µM EGCG), and 50% (100 µM EGCG) from control	Arbutin 1 mM	60% of control
Kim et al., 2019	RAW264.7 cells	Cell viability	3''Me-EGCG	90% (6,25 & 12,5 µM EGCG)	RAW264.7 cells without additional substances	100 % of control
		ROS generation DHR123(20 µM)		45% (12,5 µM)	RAW264.7 cells without additional substances	50%
		ROS generation SNP (0,25mM)		45% (12,5 µM)	RAW264.7 cells without additional substances	80%
		ROS generation DHR123 (20 µM) & SNP (0,25 mM)		45% (12,5 µM)	RAW264.7 cells without additional substances	80%
	HaCaT cells (48 hr)	DPPH scavenging activity		20% ( 6,25 µM EGCG), 50% ( 12,5 µM EGCG)	HaCaT cells without additional substances	90%
		ABTS scavenging activity		90% ( 6,25 µM and 12,5 µM EGCG )	HaCaT cells without additional substances	90%
		Cell viability H2O2 250 µM for 24 hr		80% (12,5 µM EGCG)	HaCaT cells without additional substances	60%
		NO production SNP (1,5 mM) for 24 hr		70% (12,5 µM EGCG) of control	HaCaT cells without additional substances	100%
		Cell viability of SNP (1,5 mM)		90% (12,5 µM EGCG)	HaCaT cells without additional substances	70%
		Cell viability UVB (30 mJ/cm2)	3''Me-EGCG (µM)	85% (6,5 µM EGCG & 12,5 µM EGCG)	HaCaT cells without additional substances	50% of control
		Cell viability UVB (30 mJ/cm2) LY294002 (20 µM)	3''Me-EGCG (12,5 µM)	50%	HaCaT cells without additional substances	40% of control
		Cell proliferation	EGCG 24 hr	200% ( 6,25µM EGCG), 250% (12,5 µM EGCG)	HaCaT cells without additional substances	120% of control
			EGCG 48 hr	300% (6,5 µM EGCG), 380% (12,5 µM EGCG)	HaCaT cells without additional substances	180% of control
			EGCG 72 hr	500% 6,5 µM EGCG), 600% (12,5 µM EGCG)	HaCaT cells without additional substances	300% of control
	HEK293 T cell (48 hr)	Folid increase	EGCG 24 hr, HA-AKT1	1,3 (6,25 µM EGCG), 1,2 (12,5 µM EGCG)	HEK293T cells without additional substances	1,0
					HA-AKT1 without EGCG	1,1
		Folid increase	EGCG 48 hr	1,2 (6,25 µM EGCG) , 1,4 (12,5 µM EGCG)	HEK293T cells without additional substances	1
Wagemaker et al., 2017	25 Japanese volunteers (24-55 years)	Antioxidant activity	GTP formulation	IC50% = 527 µg/ml	resveratrol	IC50% = 3765 µg/ml
		Transepidermal water loss	GTP formulation	(7 g.m2/hr)GTP has a recovered skin effect similar to resveratrol		(8g.m2/hr) showed fully recovered skin.
		Reflectance Confocal Microscopy	Treatment with vehicles, GTP, and SDS	presented desquamation, spongiosis, and inflammatory cells in the skin layers.		-
		Interleukins assay	GTP formulation	45 pg/tape		IL assay is similar to GTP was found.
Won et al., 2021	Hs68 cell	ROS Production induced by TNF-α in Hs68cells.	EGCG (1, 10, 20 µM)	EGCG suppresses cellular ROS production.	TNF-α (20 ng/ml) after 45 minutes	-
		Inhibitory MMP-1 mRNA expression and secretion in TNF-α- stimulated Hs68 dermal fibroblast		EGCG suppressed MMP-1 production and secretion, in response to the pro-inflammatory cytokine TNF-α.		MMP-1 quantity and expression in the supernatant were greater.
		Inhibitory Src-dependent ERK signalling pathway		EGCG suppresses the TNF-α-induced ERK signaling pathway and therefore affects MMP-1 expression and secretion, suppressed MEK, and Src phosphorylation		MEK and Src phosphorylation increased.
		Effect of EGCG on Phosphorylation of Akt		Increasing Akt phosphorylation		There was no increase in Akt phosphorylation.
Abotorabi et al.,2020	Human fibroblast cells	Anti-UVB effects in fibroblast cells	Green tea extracts (0-1mg/ml)	Viability cells were increased by 71% with a low concentration of green tea.	Jujube extracts (0-8 mg/ml)	85% increase in cell viability
		Evaluation of the antioxidant activity of cell culture media	Green tea extracts	The antioxidant capacity of culture media increased.	Jujube extracts	The antioxidant capacity of culture media increased.
		Effect on UVB-induced MMP-2 and -9 secretion in fibroblast cells of culture media	Green tea extracts	Demonstrated notable decreases in MMP-2 and MMP-9 at low concentrations.	Jujube extracts	Reduce MMP-2 and -9 protein in a dose-dependent on manner.
Ferzli et al., 2013	35-year-old males and females (n=32) have skin Fitzpatrick types II	facial redness	A topical antioxidant product containing GTP and caffeine	Facial redness was reduced.	A topical antioxidant product containing green tea polyphenols and caffeine with resveratrol	Facial redness was reduced.
Wisuitiprot et al., 2022	29 female volunteers who have wrinkles on their faces	Skin elasticity	Green tea cream	Increased elasticity was superior to the placebo cream	Placebo cream	-
		Melanin index		Improved after 6th week		-
		Irritation signs		No signs of irritation were found		-
Li et al., 2022	Human dermal fibroblast	Cell viability	EGCG	Viability cells increase significantly.	EGCG-niosomes	Higher viability than EGCG-free was found.
		Intracellular MDA level and the antioxidant enzyme activities		Decrease MDA level		Show MDA level much lower (0.80 ± 0.33 µmol/L/mg protein) than EGCG free
Gianeti et al., 2013	24 volunteers aged 25–40 years old have Fitzpatrick types II and III on their forearm skin	Skin moisture	6.0% [w/w] of *Camellia sinensis* [green tea] glycolic leaf extract	Increased skin moisture and reduction of roughness according to the texture	Vehicle formulation	-
Megow et al., 2017	Human skin of 32 participants	Radical scavenging properties	Benifuuki green tea	28%	Yabukita green tea	29%
Lianza et al., 2020	N-succinyl-Ala-Ala-Pro-Phe	Elastase inhibition (µg/ml)	Green tea extracts 50 µg/mL.	39% (IC50 = 100 µg/ml did not show specific results)	PMSF (241 µM)	42 µg/ ml
		Tyrosinase inhibition (µg/ml)		43% (IC50 = 66.04 ± 1.75 µg/ml).	RRO acetone extracts	IC50 of 19.26 ± 1.16 µg/ml comparable to CSI
Hong et al.,2013	ABTS radicals	ABTS scavenging activity	Normal green tea extracts	IC50 = 1.44 mg/ml	Tannase green tea extracts	(IC50 = 0.98 mg/mL) was significantly (p<0.001) higher than NGE.
	DPPH radicals	DPPH scavenging activity	Normal green tea extract	IC50 = 1.63 mg/mL	Tannase green tea extracts	(IC50 = 1.45 mg/mL) was significantly (p<0.01) higher than NGE.
	24 Korean female (30-59 years) with fine wrinkles at the lateral canthal lines	Anti-wrinkle activity	2.5 mg of formulation contains NGE	Ra = - 0.35 Rt value = -4.62 36.3% wrinkle improvement	2.5 mg of formulation contains (TGE)	Ra = - 0.89 Rt value = -9.87 63.6% wrinkle improvement

Abbreviations: 3''Me-EGCG, (−)-Epigallocatechin-3-(3''-O-methyl) gallate; ABTS, 2,2′-Azino-bis(3-ethylbenzothiazoline-6-sulphonic acid); Akt, serine/threonine kinase; CSI, *Camellia sinensis* Kuntze; DHR, dehydrorhodamine; DPPH, 2,2-diphenyl-1-picrylhydrazyl; EGCG, Epigallocatechin Gallate; ERK, extracellular signal-regulated kinase; FLG, filaggrin; GTP, Green tea polyphenols; H2O2, hydrogen peroxide; HA-AKT, hemagglutinin- serine/threonine kinase 1; HaCaT, Cultured Human Keratinocyte; HAS, hyaluronic acid synthase; HEK293T, human embryonic kidneys 293T; Hs68, human dermal fibroblast; IC50%, Half-maximal inhibitory concentration; LY294002. 2-(4-Morpholinyl)-8-phenyl-4H-1-benzopyran-4-one; MDA, Malondialdehyde; MEK, mitogen-activated protein extracellular kinase MMP, matrix metalloproteinase; NGE, normal green tea extract; NMF, natural moisturizing factor; NO, nitric oxide; Ra, arithmetic average roughness; RAW264.7, macrophage-like cell; ROS, reactive oxygen species; RRO, Rhodiola rosea L. (roots); Rt, average roughness in wrinkles formation SDS, Sodium dodecyl sulfate; SNP, sodium nitroprusside; TGE, tannase-converted green tea extract; TGM, transglutaminase; TNFα, Tumor necrosis factor alpha; UVB, ultra violet B

### In vivo studies

Data from a clinical trial using topically administered green tea extract showed wrinkle reducing effects in humans with 36.3% improvement using normal green tea extract (NGE) and 63.6% using tannase-converted green tea extract (TGE) (Hong et al., 2013). This effect can be exhibited by the chain-breaking mechanism of GTP as an antioxidant. This reaction made hydrogen atom transfer or a single electron transfer reaction or both to alkoxyl lipids and peroxyl radicals to interfere with damage caused by free radicals to cells (Lambert and Elias, 2010). 

Another randomized controlled trial also showed that the usage of green tea extract cream reduced wrinkles after four weeks of daily application (Wisuitiprot et al., 2022). Inhibition of free radicals and elastase protected the skin from damage that reduced skin elasticity, meanwhile, the increased cell proliferation could benefit the skin during the healing process and increase skin elasticity. The effects shown off by EGCG supplementation would bring benefits for slowing the skin aging process (Balasubramanian and Eckert, 2007; Farage et al., 2008; Kim et al., 2018, 2019; Lianza et al., 2020). 

A trial in humans also ensured this effect by showing benefits in melanin index reduction after the application of green tea extract cream for six weeks (Wisuitiprot et al., 2022). Reduction of transepidermal water loss from the skin was also confirmed in a randomized controlled trial. This study suggested that the usage of GTP extract on the skin reduced water loss from 9 g/m^2^/hr to 7 g/m^2^/hr (Wagemaker et al., 2017). This effect might be detected because of the EGCG effect on natural moisturizing factors (NMF) gen syntheses such as filaggrin (FLG), transglutaminase-1, hyaluronic acid synthase (HAS-1), and HAS-2 (Kim et al., 2018).

## Discussion

Although EGCG shows broad benefits for the skin, application of EGCG in dermatologic and cosmetics fields is still scarce (Frasheri et al., 2020). Various factors such as the presence of other substances with similar properties, concentration, as well as most notably pH and temperature can lead to EGCG degradation (Friedman et al., 2009). These various factors decrease EGCG stability through oxidation and epimerization processes (Krupkova et al., 2016). Besides all of those negative factors, some studies suggested that the combination of EGCG with vitamins C and E, particularly vitamin C, can increase the stability of catechin from green tea extract (Intra and Kuo, 2007; Scalia et al., 2013).

There is another problem with using EGCG as a cosmetic ingredient. The stratum corneum of the skin causes the absorption of exogenous EGCG to be inhibited and limits the penetration of the substance into the dermis. One carrier that can be used to overcome the absorption problem is niosomes, a nanocarrier consisting of non-ionic surfactants and cholesterol (Chen et al., 2019; Li et al., 2022). In testing the penetration of drugs with niosomes, one of the most important things to assess is the entrapment efficiency (EE), where the higher the EE, the higher the therapeutic effect of the drug. In an experiment, the EGCG-niosome mixture achieved an EE of 53.05 ± 4.46% with the drug distribution type starting with an initial rapid encapsulation breakdown followed by a slow release later. This result is interesting because rapid release at the beginning of the phase can increase drug penetration and slow release can maintain the drug for a longer duration in the skin so that drug application can be done less frequently. The mixture of EGCG with niosomes produced particles of 235.4±15.64 nm and a zeta potential of -45.2±0.03 mV. At sizes less than 300 nm, particles can cause excessive transdermal drug transport. Particles are considered stable when they have a zeta potential below -30 mV, but in the EGCG mixture, it is necessary to add dihexadecyl phosphate (DCP) so that the zeta potential of EGCG-niosomes can reach a stable state (Jacobs and Müller, 2002; Chen et al., 2019; Li et al., 2022).

In this study, the positive effects of green tea, green tea polyphenols, and EGCG extract show many benefits related to the skin aging process such as inhibition of wrinkles, increased cell proliferation, inhibition of reactive oxygen species production, scavenging effect against free radicals, increased elasticity, and decreased skin melanin index. However, the use of green tea still requires further research due to the unstable nature of green tea. The use of a carrier for green tea molecules shows promising effects as it increases the stability and penetration of EGCG molecules into the skin. Therefore, further research on the use of molecular carriers and their application to human skin is of interest. 
